# Mental health after unintended pregnancy: insights from a context with available abortion care

**DOI:** 10.1007/s00737-026-01745-8

**Published:** 2026-07-04

**Authors:** Wieke Y Beumer, Tessa J Roseboom, Jenneke van Ditzhuijzen

**Affiliations:** 1 Dept. of Gynaecology and Obstetrics, Amsterdam UMC location University of Amsterdam, Meibergdreef 9, Amsterdam, Netherlands; 2 Dept. of Epidemiology and Data Science, Amsterdam UMC location Vrije Universiteit Amsterdam, De Boelelaan 1117, Amsterdam, Netherlands; 3 Interdisciplinary Social Sciences, Utrecht University, Utrecht, Netherlands; 4 Amsterdam Reproduction and Development research institute, Amsterdam, Netherlands

**Keywords:** Unintended pregnancy, Unwanted pregnancy, Unplanned pregnancy, Abortion, Continued to term, Mental health, Depression, Anxiety, Social support

## Abstract

**Purpose:**

To investigate how unintended pregnancy (UP) is associated with the occurrence of mental health (MH) problems one year later among womxn, while also considering pregnancy outcome (abortion/continuation), co-occurring risks and protective factors. We examine this in the Dutch context, given available abortion care up to 24 weeks gestation and high diversity in people’s pregnancy intentions. We hypothesized that MH problems are more prevalent among those whose pregnancy was more unintended.

**Methods:**

Data from the Dutch BluePrInt study included both womxn who had an abortion (AB, *n* = 152) and who continued their UP (CT, *n* = 212). They filled out an online survey shortly after their abortion (AB) or around 14 weeks gestation (CT), and at follow-up one year later. Key variables included pregnancy intendedness (continuously measured), anxiety/depression symptoms (measured with a screening tool based on the CIDI), social- and partner support, abuse experiences, UP-related variables (difficulty deciding, uncertainty, pressure, and negative emotions) and socioeconomic position.

**Results:**

26.5% developed MH symptoms one year after their UP, with no significant differences between AB and CT groups. Pregnancy intendedness and -outcome (abortion vs. continuation), as well as their interaction, were not related to MH symptoms one year later. Previous MH was the strongest predictor of MH risks (OR: 3.54), while perceived social support from family/friends was associated with a lower risk (OR: 0.66).

**Conclusions:**

In a context where abortion care is available, pregnancy intendedness and -outcome do not increase people’s risks of MH symptoms one year after experiencing an UP. Instead, previous MH and support from family/friends are important for MH after experiencing an UP. Positioning UP as inherently problematic is not warranted. Policy and practice should center people’s lived reproductive experiences and care needs.

**Supplementary Information:**

The online version contains supplementary material available at https://doi.org/10.1007/s00737-026-01745-8.

## Introduction

Having an abortion does not increase the risk of mental health (MH) problems (APA 2008; Charles et al. 2008; Foster et al. 2015; Howard et al. 2025; Littell et al. 2024; National Collaborating Centre for Mental Health, 2011). In contrast, carrying an unintended pregnancy (UP) to term may increase this risk (Gipson et al. 2008): early motherhood is a stressful period (McConachie et al. 2008; Razurel et al. 2013), perhaps more so if the baby is the result of an UP (Harris et al. 2014; McNamara et al. 2022). However, the potential MH impact of continuing an unintended pregnancy is less well understood, as evidence is mixed (Gipson et al. 2008), and particularly scarce among those who choose to continue their UP rather than being denied abortion. In this context, the Netherlands offers a unique case: abortion is free and available until 22 weeks of gestation (Holten et al. 2021; IGJ, 2025), hence very few people are denied abortions. Around 28% of Dutch pregnancies are unintended; 53% of these are carried to term (Bearak et al. 2022).

Not only the UP itself, but also the circumstances that make a pregnancy more unintended, could result in MH problems. This aligns with the stress and coping theory (Lazarus 1984), which posits that people’s responses to potentially stressful life events - such as UP (Major et al. 2008) - are shaped by interactions between a person and their environment. In this framework, an UP does not inevitably lead to adverse MH outcomes; rather, it depends on contextual and personal factors. Yet most research on MH consequences of UP did not adequately control for these factors (Auerbach et al. 2023; Hill et al. 2019).

A key factor to consider is people’s history of MH problems, which prior research has consistently identified as a strong predictor of MH problems following an UP (Beumer et al. 2023; Littell et al. 2024; Schonewille et al. 2024; Steinberg et al. 2014; van Ditzhuijzen et al. 2013). Other important factors include co-occurring risks, such as socioeconomic position (SEP) and experiences of abuse, which increase the risk of both UP (Goossens et al. 2016; Maxson and Miranda 2011) and MH problems (Allen et al. 2014; Boden et al. 2009; De Graaf et al. 2010; Steinberg and Tschann 2013; Van der Heide et al. 2013). Conversely, factors like social support, including partner support, can protect against MH problems after an UP (Bahk et al. 2015; Chan 2021; Kazemi et al. 2021; Lewinsohn et al. 2018; van Ditzhuijzen et al. 2017). Additionally, UP-related factors (such as difficulty deciding, uncertainty, pressure, and negative emotions surrounding the decision for abortion or continuation of the pregnancy) may be related to subsequent MH problems, but findings have been mixed (Broen et al. 2006; Fergusson et al. 2009; van Ditzhuijzen et al. 2017), and these factors have not yet been studied in people continuing an UP.

Prior research has mainly compared MH problems of people with UPs to those with intended pregnancies. However, dichotomizing pregnancies as either intended or unintended oversimplifies a multidimensional, continuous construct (Barrett et al. 2004). Moreover, it is difficult to disentangle the effects of pregnancy outcome (abortion vs. continuation) from pregnancy intendedness. Although these are strongly correlated, they are inherently different: not all people with highly unintended pregnancies choose for abortion, nor do all people with highly intended pregnancies continue these (Sprenger et al. 2025). Disentangling pregnancy outcomes from intentions is important, as those who continue a pregnancy that is very unintended may be particularly vulnerable to MH consequences (David 2006; Foster et al. 2015).

The current study investigates how UP is associated with the occurrence of MH symptoms one year later among womxn^1^ in the Netherlands, while also taking into account the pregnancy outcome (abortion/continuation). We conceptualized UP as a continuous construct, indicating higher or lower levels of intendedness. We are especially interested in how pregnancy intendedness and -outcome interact, as we hypothesize that people who continue a highly unintended pregnancy may face increased risks of MH problems. Additionally, we aim to assess the impact of potential co-occurring risks and protective factors. We hypothesize that a history of MH problems, experiences of abuse, lower SEP, and more adverse decision-related factors (including difficulty deciding, uncertainty, pressure, and negative emotions) may increase the risk of MH symptoms. Conversely, we expect that more perceived support from a partner, family and friends may reduce this risk.

## Methods

### Study design and procedure

The BluePrint study is a prospective study into UP in the Netherlands. Data came from the first (March 15 – October 22, 2022) and second wave (January 13 – October 19, 2023). The current sample included womxn who chose abortion (AB) or continuation of their UP (CT). AB respondents were recruited from nine abortion clinics across the Netherlands. More than 90% of abortions are performed in these clinics (until 22 weeks gestation) (Holten et al. 2021; IGJ 2025). Recruitment of CT respondents took place at 83 midwifery clinics nationwide, around eight weeks gestation.

People could sign up by scanning a QR code, either while waiting or during their consultation session. Additional CT respondents were recruited through an online mailing via their midwife. The QR code linked to a webpage to sign up for the study. This included an information letter and video. Respondents provided written informed consent.

To avoid any potential decision-making interference, people could only participate after having an abortion or deciding to continue their pregnancy. They were eligible if they were older than 15 years. CT respondents qualified if they met at least two of the following: they weren’t trying to conceive, felt the timing was wrong, or had mixed or negative feelings about the pregnancy.

Respondents received a link to an online survey via email. Most respondents completed the surveys on their phones. The first survey took 15 min to complete. The follow-up survey was administered approximately one year later, which took around 10 min to complete. Respondents received two 10-euro gift cards for completing the two surveys.

The survey was available in Dutch. The survey was pretested with two experience experts, a linguist, sexologist, epidemiologist, abortion physician, and midwife.

### Study population

At baseline, 690 womxn filled out the survey (260 AB, 430 CT). One year later, 364 respondents (52.8%) filled out the follow-up (152 AB, 212 CT). Attrition analysis showed that those lost to follow-up were significantly younger, less often unemployed, multiparous and religious (Table S1). They also had experienced MH symptoms or abuse significantly less often, and they experienced more difficulty deciding for abortion or continuation of their pregnancy. There were no differences in levels of pregnancy intendedness.

In our main analyses, we excluded 39 respondents who reported first-time MH symptoms in the six months prior to our baseline survey. These 39 participants were excluded to maintain proper temporal ordering for causal inference, as we cannot ascertain what came first: MH symptoms or UP. The final sample (*n* = 325) therefore consisted of respondents who either had never experienced MH symptoms at baseline or had only experienced such symptoms in the more distant past (> 6 months prior to baseline).

### Measures

- **Symptoms of MH problems** were conceptualized as having symptoms of anxiety or depression. We screened for symptoms with the Lifetime Depression Assessment Self-Report (Bot et al. 2017; Fedko et al. 2020): an online self-assessment tool that is largely based on the Composite International Diagnostic Interview (Kessler et al. 2006). We included three items on symptoms of major depressive disorder and eight items on anxiety disorders (five on social phobia and three on generalized anxiety disorder). Not all participants answered all items; for example, those who answered ‘no’ to the initial screening item per disorder did not receive the follow-up items. Cutoff scores followed the CIDI screener (Kessler et al. 2006). At baseline, we screened for lifetime history of MH symptoms (1 item per disorder). Respondents specified the timing of symptom occurrence: more than a year ago, between six months and a year ago, or within the past six months. At follow-up, the reference period was the time since baseline.
- **Pregnancy intentions.** We adapted the London Measure of Unplanned Pregnancy (LMUP) (Barrett et al. 2004) to the Dutch context. The LMUP includes six items on contraceptive use, preconceptional behavior, partner communication, timing, desire for parenthood, and intention to become pregnant. Although we made substantial modifications to the measure (Sprenger et al. 2025), it demonstrated acceptable reliability (current study’s Cronbach’s α = 0.72). Each item was scored 0 to 2, with a total score of 0 to 12; higher scores indicated higher levels of intendedness.
- **Perceived social support from family/friends and partner.** The Multidimensional Scale of Perceived Social Support (MSPSS) (Zimet et al. 1988) subscale consisted of five items on a 5-point scale. A mean score was calculated (Cronbach’s α = 0.96). Another MSPSS subscale measured perceived social support from a romantic partner (Cronbach’s α = 0.95). People without a relationship were given a neutral score.
- **Decision related variables.** These were all measured on a five-point scale. First, we assessed people’s certainty with their decision for abortion or continuation of the pregnancy (right decision, satisfied with decision, and ‘*I would have made the same decision today*’ (van Ditzhuijzen et al. 2017). A mean score was calculated (Cronbach’s α = 0.76). Further, we assessed both difficulty and perceived pressure to make a certain decision with one item. Lastly, respondents indicated their agreement with statements reflecting post-decision negative emotions, including guilt, emptiness, mourning, and sadness. A mean score was calculated (Cronbach’s α = 0.87).
- **Experiences with abuse.** Participants indicated whether they experienced mental or physical childhood abuse (< 16 years old). Further, participants indicated having experienced sexual victimization in their lifetime.
- **Socioeconomic variables.** Several indicators of socioeconomic position (SEP) were included. To assess subjective SEP, respondents were presented a visual ladder ranging from 0 to 10, and rated their socio-economic position in relation to their community (Adler et al. 2000; Zhao et al. 2023). We also measured unemployment, educational level and birth country.

### Analyses

Statistical analyses were performed in R Studio by WB. After performing the descriptive analyses of potential predictor variables, we calculated the at-risk population to ensure a correct temporal order of events. We excluded participants (*n* = 39) who experienced first-time MH symptoms in the 6 months prior to baseline. As a sensitivity analysis, we also repeated all analyses with these participants reinstated (Table S2).

We assessed the linearity assumption for all continuously measured predictors by inspecting their scatterplots, and found no evidence of non-linearity. To avoid the information loss introduced by categorization, we modeled these variables in continuous form. Multicollinearity was examined and not found to be problematic (tolerance > 0.1; VIF < 10). Finally, we included an interaction term between pregnancy intendedness and outcome (continuation vs. abortion) to test whether the risk of MH symptoms was particularly elevated among those who continued a highly unintended pregnancy.

Bivariate logistic regression analyses were performed to examine associations between each predictor and the occurrence of MH symptoms at follow-up. Variables that were significant at *p* < .10 in the bivariate analyses were included in the multivariate logistic regression models. In the multivariate analyses, background variables with *p* > .10 were removed using backward stepwise elimination. After each removal, model fit was reassessed to determine whether the exclusion improved the model. If model fit did not significantly worsen, the variable was excluded, in line with our aim for parsimony.

From an etiological perspective, we aimed to distinguish between incident and recurrent MH symptoms at follow-up, to capture the onset and re-emergence of MH symptoms in response to UP (Rothman et al. 2024). Womxn were considered at risk of incident MH symptoms if they had no previous history of MH symptoms; those with a history of MH symptoms were considered at risk of recurrent MH symptoms. Thus, we tested interactions between MH history and each significant predictor to assess differences between onset and recurrence of MH symptoms. As none of these interactions were statistically significant, we analyzed overall MH symptom occurrence rather than conducting separate analyses.

At each previously described step, we analyzed both the combined occurrence and the separate outcomes of anxiety and depressive symptoms. All tests were two-sided, with statistical significance set at *p* < .05.

## Results

### Sample characteristics

At baseline, participants were between 16 and 47 years old. Womxn who continued their UP (CT) reported higher levels of intendedness compared to womxn who had an abortion (AB) (Table 1). Participants who chose to continue a pregnancy that was highly unintended did not significantly differ on background variables from other CT participants, although those who continued a very unintended pregnancy reported significantly more decision difficulty. Furthermore, compared to AB participants, those who continued a very unintended pregnancy were significantly older, more often in a relationship or unemployed, experienced less decision difficulty and less negative emotions after their decision.

**Table 1:** Selected baseline sample characteristics

Variable	Total	Abortion	Continued, somewhat unintended (LMUP 4–12) ^a^	Continued, highly unintended (LMUP 0–3) ^a^
	*n* = 364	*n* = 152	*n* = 160	*n* = 52
Unintended pregnancy				
Pregnancy intendedness ^a^ (0–12, higher = more intended) (*M*,* SD)*	4.02 (2.75)	2.06 (1.81)	6.43 (1.84)*	2.26 (0.83)
Sociodemographic				
Subjective SEP (0–10) (*M*,* SD*)	5.94 (2.00)	5.50 (2.07)	6.27 (1.80)	6.15 (2.23)
Employment status (N, %)		**		
Unemployed	44 (12.1)	16 (10.5)	20 (12.5)	8 (15.4)
Only studying	97 (26.6)	26 (17.1)	50 (31.3)	21 (40.4)
Employed	220 (60.4)	107 (70.4)	90 (56.3)	23 (44.2)
Educational level (N, %)				
Practical	191 (52.5)	92 (60.5)	70 (43.8)	29 (55.8)
Theoretical	172 (47.3)	59 (38.8)	90 (56.3)	23 (44.2)
Born in the Netherlands (yes) (N, %)	343 (94.2)	144 (94.7)	151 (94.4)	48 (92.3)
Age (*M*, SD)	29.7 (6.06)	28.56 (7.23)*	30.21 (5.08)	31.10 (4.77)
15–20	29 (7.7)	23 (15.1)	5 (3.1)	1 (1.9)
21–39	317 (87.1)	118 (77.6)	149 (93.1)	50 (96.2)
> 39	17 (4.7)	10 (6.6)	6 (3.8)	1 (1.9)
Religious (yes) (N, %)	93 (25.5)	27 (17.8)	51 (31.9)	15 (28.8)
Relation status (N, %)		**		
Single	30 (8.2)	20 (13.2)	7 (4.4)	3 (5.8)
In a relationship, not cohabiting	88 (24.2)	59 (38.8)	20 (12.5)	9 (17.3)
Cohabiting	238 (65.4)	69 (45.4)	129 (80.6)	40 (76.9)
Parity (multiparous) (N, %)	156 (42.9)	55 (36.2)	66 (41.3)	35 (67.3)
Abuse				
Experience with sexual violence (N, %)	80 (22.0)	35 (23.0)	35 (21.9)	10 (19.2)
Experience with physical/mental abuse during youth (N, %)	62 (17.0)	26 (17.1)	27 (16.9)	9 (17.3)
Social support				
Perceived support family/friends (0–4) (M, SD)	3.21 (0.81)	3.14 (0.86)	3.34 (0.70)	3.07 (0.91)
Perceived partner support (0–4) (M, SD)	3.01 (1.21)	2.90 (1.39)	3.14 (1.07)	3.02 (1.05)
Decision related variables				
Perceived pressure (0–4) (M, SD)	0.43 (0.73)	0.40 (0.73)	0.46 (0.76)	0.56 (0.73)
Difficulty (0–4) (M, SD)	1.22 (1.28)	1.88 (1.39)***	0.66 (0.96)*	1.05 (1.05)
Certainty (0–4) (M, SD)	3.39 (0.71)	3.29 (0.68)	3.53 (0.71)	3.43 (0.66)
Negative emotions (0–4) (M, SD)	0.95 (1.06)	1.55 (1.15)***	0.48 (0.73)	0.70 (0.84)

*Notes.* ^a^ Pregnancy intentions were measured with the Dutch Adaptation of the Londen Measure of Unplanned Pregnancy (DA-LMUP). *Mean or distribution differs from that for the continued, unintended group at *p*<.05; **Mean or distribution differs from that for the continued, unintended group at *p*<.01; ***Mean or distribution differs from that for the continued, unintended group at *p*<.001

### Occurrence of Symptoms of MH problems

Of the 325 womxn, 26.5% experienced MH symptoms one year after their UP. Specifically, of the 143 womxn with no previous MH symptoms, 25.9% developed symptoms (i.e., incidence). Of the 182 womxn with previous MH symptoms, 33.0% experienced symptoms (i.e., recurrence). MH symptom incidence and recurrence did not differ significantly between the AB and CT groups (Table 2).

**Table 2:** Number of respondents at risk of incident or recurrent symptoms of mental health problems a year after experiencing an unintended pregnancy, and percentage experiencing symptoms of mental health problems, by type of disorder

	Total (*n* = 325)		Abortion (*n* = 135)		Continued, somewhat unintended (*n* = 142)		Continued, highly unintended (*n* = 48)	
	*n* at risk	% with MH symp T1	*n* at risk	% with MH symp T1	*n* at risk	% with MH symp T1	*n* at risk	% with MH symp T1
Incident or recurrent T1								
Major depression	325	18.5	135	18.5	142	18.3	48	18.8
Anxiety	325	17.8	135	15.6	142	18.3	48	22.9
Any	325	26.5	135	24.4	142	27.5	48	29.2
Incident T1								
Major depression	203	9.9	92	13.0	85	9.4	26	0.0
Anxiety	193	10.9	87	8.0	80	12.5	26	15.4
Any	143	25.9	69	23.2	55	30.9	19	21.1
Recurrent T1								
Major depression	122	32.8	43	30.2	57	31.6	22	40.9
Anxiety	132	28.0	48	29.2	62	25.8	22	31.8
Any	182	33.0	66	28.8	87	34.5	29	37.9

*Notes*. Womxn were considered at risk of *incident* symptoms of MH problems if they had no history of experiencing these symptoms. They were considered at risk of *recurrent* symptoms of MH problems if they had a history of experiencing these symptoms

In the bivariate analyses, pregnancy intendedness, and -outcome (continued vs. abortion) were not significantly related to MH symptoms at follow-up (Table 3). Further, higher subjective SEP was associated with a decreased risk of MH symptoms at follow-up; being foreign-born, unemployed, and having experienced abuse were associated with a higher risk. History of MH symptoms, perceived social support from both family/friends and a partner, decision pressure, and negative emotions were also significantly associated with increased risk. Education, cohabitation, age, parity, religiosity, decision difficulty and certainty were not included in the multivariate models due to non-significant bivariate associations (*p* > .10).

**Table 3:** Odds ratios (and 95% confidence intervals) from bivariate and multivariate logistic regression analyses examining the associations between selected characteristics and the occurrence of any symptoms of a mental health problem (depression and anxiety) at follow-up

	Bivariate	Multivariate
Pregnancy intendedness (higher score = more intended)	1.03 (0.95–1.13)	0.97 (0.77–1.23)
Pregnancy outcome (0 = abortion)	1.15 (0.70–1.92)	1.10 (0.36–3.34)
Pregnancy intendedness x Pregnancy outcome	-	1.04 (0.79–1.38)
Sociodemographic		
Higher subjective SEP	0.87 (0.77–0.98)*	0.87 (0.76–1.01) •
Born outside the Netherlands	3.06 (1.02–9.22)*	na
Unemployed	3.38 (1.68–6.79)***	1.97 (0.86–4.44)
Practically educated (vs. theoretically)	0.79 (0.48–1.30)	na
Cohabiting	1.05 (0.62–1.81)	na
Age	1.02 (0.98–1.06)	na
Multiparous	1.25 (0.76–2.06)	na
Religious	1.42 (0.81–2.47)	na
Abuse		
Experiences with mental/physical abuse during youth	3.20 (1.69–6.03)***	1.95 (0.94–4.01) •
Experiences with sexual abuse in lifetime	1.96 (1.11–3.45)*	na
History of mental health symptoms	3.96 (2.28–7.14)***	3.54 (1.92–6.85)***
Social support		
Support from family/friends	0.60 (0.43–0.84)**	0.66 (0.45–0.95)*
Support from partner	0.79 (0.65–0.96)*	na
Decision related		
Perceived pressure	1.47 (1.03–2.09)*	1.43 (0.97–2.12) •
Difficulty	1.01 (0.82–1.23)	na
Certainty	1.22 (0.85–1.82)	na
Negative emotions	1.31 (1.03–1.67)*	na

*Notes.* • *p*<.10, *p<.05, ** *p*<.01, *** *p*<.001. Na = not applicable. Variables were not included in the multivariate model if they were not significantly related to the outcome in bivariate analyses, or the model fit significantly approved when the variable was excluded from the multivariate model. All characteristics were measured at baseline

In multivariate analyses, being born outside the Netherlands, sexual abuse experiences, partner support, and decision related negative emotions were excluded using backwards stepwise elimination. In the final multivariate model, pregnancy intendedness and outcome -, as well as their interaction, were not associated to MH symptoms at follow-up (Table 3). MH history was the strongest predictor of MH symptoms at follow-up (OR: 3.54; CI = 1.92–6.85). Further, perceived social support from family/friends was significantly related to lower MH risks (OR: 0.66, CI = 0.45–0.95).

We ran sensitivity analyses using the full sample, including the 39 participants with first-time MH symptoms in the six months prior to baseline. Results closely mirrored the main multivariate findings, with MH history as the most important predictor of MH symptoms at follow-up (Table S2). In comparison to the main analyses results, perceived partner support became a significant predictor of MH symptoms at follow-up.

### Occurrence of Anxiety and Depressive Symptoms Separately

As in the overall analysis, levels of pregnancy intendedness and pregnancy outcome, as well as their interaction, were not associated with the risk of anxiety/depression symptom occurrence at follow-up (Table S3a and b). Having a history of anxiety/depression symptoms was the strongest predictor of experiencing those symptoms at follow-up (Anxiety OR: 2.88, CI: 1.54–5.54; Depression OR: 5.37, CI = 2.76–10.90). In contrast to the overall analysis, social support from family/friends was not significantly associated with the risk of anxiety symptoms, although it was significantly associated with a lower risk of depressive symptoms (OR: 0.66, CI = 0.44–0.99). People who were unemployed had higher odds of reporting anxiety symptoms at follow up (OR: 2.52, CI: 1.11–5.54). Also in contrast to results of the overall analysis, people who experienced more negative emotions regarding their decision at baseline, reported more anxiety symptoms at follow-up (OR: 1.55, CI = 1.10–2.20), although only marginally significant for depression (OR: 1.37, CI = 0.95–1.97).

## Discussion

Although ample studies concluded that abortion does not increase the risk on MH problems (APA, 2008; Charles et al. 2008; Foster et al. 2015; Howard et al. 2025; Littell et al. 2024; National Collaborating Centre for Mental Health, 2011), up to now less was known about the risks of continuing an UP in a context with available abortion care. Additionally, a study that simultaneously considers both pregnancy intendedness (measured continuously) and - outcome (abortion vs. continuation) had not yet been conducted. Our results did not support our hypothesis that people who continue a pregnancy that was very unintended would be at elevated risk of experiencing MH symptoms: neither pregnancy intendedness, outcome (abortion vs. continuation), nor their interaction predicted MH symptoms one year later. Instead, people who had a history of experiencing MH symptoms, they had higher risks of MH symptoms a year after their UP. Further, people who perceived more social support from family/friends had lower risks of MH symptoms a year later.

In the Netherlands, abortion is available until 22 weeks of gestation (Holten et al. 2021; IGJ, 2025), hence very few womxn are denied abortion care. This context may help explain why we did not observe elevated MH risks following an UP: most participants in our study were likely able to make reproductive choices that suited their personal circumstances. In contrast, previous research has shown that people who are denied abortions do face higher MH risks, although these risks diminished over time (Foster et al. 2015; Harris et al. 2014).

A key finding of our study is that prior MH symptoms were the strongest and most consistent predictor of MH symptoms a year after experiencing UP, confirming earlier research (Beumer et al. 2023; Littell et al. 2024; Schonewille et al. 2024; Steinberg et al. 2014; van Ditzhuijzen et al. 2013). People with a MH history are more likely to experience UP (Schonewille et al. 2022), a pattern reflected in our sample, where MH symptom prevalence exceeded that of the general Dutch population (Ten Have et al. 2023). Having a history of MH symptoms may predispose womxn to future MH problems, regardless of whether they experience UP. Thus, our results strongly support evidence that MH history explains the associations often found between UP -and abortion in particular- and MH outcomes in earlier studies (Howard et al. 2025; Littell et al. 2024).

Further, the finding that people who perceived greater social support had lower risks of MH symptoms, in particular depressive symptoms, is consistent with previous studies. This highlights that social support is critical to people’s MH, particularly when coping with potential challenging life events related to UP (Gray 2014; Herbell and Zauszniewski 2019; Kimport et al. 2011; Razurel et al. 2013; Xu et al. 2025). Perceived support from family and friends was a stronger predictor of MH symptoms than partner support. Most participants were in stable, often cohabiting relationships, which may have limited variability in perceived partner support. Thus, partner support likely remains important for people’s MH following an UP (Bahk et al. 2015; Kazemi et al. 2021; van Ditzhuijzen et al. 2017).

Additionally, both experiences with childhood abuse and lower SEP were associated with increased MH risks, although only marginally significant in multivariate models. These results might reflect a broader pattern of structural inequalities explaining why some people experience MH problems, and others do not. Limited social support, lower SEP, and abuse experiences are consistently linked to increased risks of MH problems in the general population (Allen et al. 2014; Boden et al. 2009; De Graaf et al. 2010; Steinberg and Tschann 2013; Van der Heide et al. 2013; Xu et al. 2025), reconfirming that UP may not pose unique MH risks.

Notably, none of the UP decision-related variables (difficulty, certainty, pressure and negative emotions regarding people’s decision to have an abortion or continue their pregnancy) were related to MH symptoms in multivariate models, confirming earlier Dutch findings into abortion, but not UP in general (van Ditzhuijzen et al. 2017). While negative emotional reactions after the decision showed a significant association with depressive symptoms when analyzed separately, our results indicate that pre-existing MH symptoms are the primary drivers of subsequent MH symptoms. Although MH history may affect how UP and decision-making are experienced (van Ditzhuijzen et al. 2015), these decision-related factors are not associated with MH symptoms after experiencing UP once other key factors are accounted for.

Our study had several limitations. Our sample predominantly consisted of highly educated, Netherlands-born womxn who were in a stable relationship, limiting generalizability to more diverse populations. Additionally, participants who had an abortion differed from those who continued their pregnancy on several sociodemographic characteristics. These differences likely reflect factors that may have influenced their pregnancy decision, such as being in a stable relationship or religiosity ( Beumer et al. 2025). Further, MH symptoms were assessed using a self-reported screening tool, which might not fully capture clinical disorders. However, we did ask about clinically diagnosed disorders with a validated tool, and this did not yield a different picture compared to previous studies. Additionally, although studies on UP are prone to drop-out (APA, 2008), the relatively high dropout rate may have led to an underestimation of MH symptoms at follow-up, despite no baseline differences in MH symptoms.

Future research could benefit from including other constructs related to people’s desire to delay or avoid pregnancy when examining MH consequences. In this study, we relied on retrospectively measured pregnancy intentions, which often shift over time to align with the chosen outcome (Rocca et al. 2019). Measures such as pregnancy acceptability (Aiken et al. 2016) or supportability (Macleod 2016) may more accurately capture factors linked to later MH problems.

Further, our finding that people’s MH history is the strongest predictor of their MH symptoms after an UP, highlights the need for future research to account for it to avoid misleading conclusions (Littell et al. 2024).

In sum, in a context where abortion care is available, pregnancy intendedness and -outcome do not increase people’s MH symptoms one year after experiencing an UP. Instead, previous MH and social support from family/friends are important for people’s MH after an UP. Given the evidence that denying abortions has bigger effects on MH (Foster et al. 2015; Harris et al. 2014), our findings highlight that reproductive autonomy is crucial for people’s MH as well. These personal and contextual factors are relevant for the MH of people across the broader population. We recommend that policy and practice move beyond positioning UP as an inherently problematic event, and more fully embrace the complexity and nuance of people’s reproductive experiences.

## Supplementary Information

Below is the link to the electronic supplementary material.

Supplementary Material 1 (PDF 229 KB)

## Data Availability

No datasets were generated or analysed during the current study.

## Footnotes

1 We did not assess participants’ gender identity. Therefore, this term refers to individuals with the capacity for pregnancy, including transgender men and nonbinary people.
