# Supplementary material for: Mental health after unintended pregnancy: insights from a context with available abortion care
Source: Arch Womens Ment Health. 2026 Jul 4;29(4):104. doi: 10.1007/s00737-026-01745-8 (PMC13331885; doi:10.1007/s00737-026-01745-8)
Supplement: Supplementary file 1 — Supplementary Material 1 (PDF 229 KB) [file 737_2026_1745_MOESM1_ESM.pdf]

Supplementary - Mental health after unintended pregnancy: Insights from a context with available abortion care

Table S1. Attrition analyses examining differences between participants who dropped out and those retained in the study.

Variable	Drop-outs after T0<br>n = 326	Responders (T0 and T1)<br>n = 364	Difference test
**Unintended pregnancy**			
Unintended pregnancy was continued (N, %)	218 (66.9)	212 (58.2)	
Pregnancy intendedness [a] (higher = more intended) (*M, SD)*	3.97 (2.77)	4.02 (2.75)	
**Sociodemographic**			
Unemployed (N, %)	21 (6.4)	44 (12.1)	**
Theoretically educated (N, %)	172 (52.8)	191 (52.5)	
Born in the Netherlands (N, %)	302 (92.6)	343 (94.2)	
Age (*M,* SD)	26.93 (5.46)	29.7 (6.06)	***
Religious (N, %)	61 (18.7)	93 (25.5)	*
Relation status (N, %)			
Single	31 (9.5)	30 (8.2)	
In a relationship, not cohabiting	62 (19.0)	88 (24.2)	
Cohabiting	219 (67.2)	238 (65.4)	
Parity (multiparous) (N, %)	79 (24.2)	156 (42.9)	***
**Abuse**			
Experience with sexual violence (N, %)	42 (12.9)	80 (22.0)	***
Experience with physical/mental abuse during youth (N, %)	33 (10.1)	62 (17.0)	**
**History of symptoms of mental health problems**	91 (27.9)	201 (55.2)	***
**Social support**			
Perceived support family/friends (0-4) (M, SD)	3.02 (1.17)	3.21 (0.81)	
Perceived partner support (0-4) (M, SD)	2.64 (1.42)	3.01 (1.21)	*
**Decision related variables**			
Perceived pressure (0-4) (M, SD)	0.44 (0.73)	0.43 (0.73)	
Difficulty (0-4) (M, SD)	1.65 (1.35)	1.22 (1.28)	***
Certainty (0-4) (M, SD)	3.42 (0.72)	3.39 (0.71)	
Negative emotions (0-4) (M, SD)	0.87 (1.02)	0.95 (1.06)	

Notes. *p<.05, ** p<.01, *** p<.001.

Table S2. Odds ratios (95% CIs) from the multivariate logistic regression analysis of factors associated with mental health symptoms at follow-up, including reinstated participants with first-time symptoms prior to baseline (n = 364; reinstated n = 39).

	**Bivariate**	**Multivariate**
Levels of UP (higher score = more intended)	1.03 (0.95 – 1.12)	1.08 (0.86 - 1.36)
Pregnancy outcome (0 = abortion)	1.10 (0.69 – 1.74)	1.21 (0.40 – 3.65)
Pregnancy intendedness x Pregnancy outcome	-	0.93 (0.71 – 1.21)
**Sociodemographic**		
Higher subjective SEP	0.86 (0.77 – 0.96)**	0.89 (0.77 - 1.02) •
Born outside the Netherlands	3.02 (1.18 – 7.83)*	na
Unemployed	2.88 (1.51 – 5.49)**	1.63 (0.72 - 3.63)
Practically educated (vs theoretically)	0.73 (0.46 – 1.15)	na
Cohabiting	0.86 (0.53 - 1.41)	na
Age	1.01 (0.97 - 1.05)	na
Multiparous	1.18 (0.74 – 1.86)	na
Religious	1.68 (1.01 – 2.77)*	na
**Abuse**		
Experiences with mental/physical abuse during youth	3.65 (2.08 – 6.44)***	2.44 (1.23 - 4.82)**
Experiences with sexual abuse in lifetime	1.00 (-0.98 – 1.02)	na
**History of mental health symptoms**	2.56 (1.59 – 4.19)***	2.93 (1.65 – 5.35)***
**Social support**		
Social support of family/friends	0.60 (0.45 – 0.79)***	0.68 (0.48 – 0.97)*
Social support of partner	0.58 (0.41 – 0.80)***	0.64 (0.43 – 0.96)*
**Decision related**		
Perceived pressure	1.00 (0.97 – 1.02)	na
Difficulty	1.02 (0.85 – 1.22)	na
Certainty	1.01 (0.99-1.02)	na
Negative emotions	1.01 (0.99 – 1.02)	na

Notes. • *p*<.10, *p<.05, ** p<.01, *** p<.001. Na = not applicable. Variables were not included in the multivariate model if they were not significantly related to the outcome in bivariate analyses, or the model fit significantly approved when the variable was excluded from the multivariate model. All characteristics were measured at baseline

Table S3a. Odds ratios (and 95% confidence intervals) from bivariate and multivariate logistic regression analyses examining the associations between selected characteristics and the risk on occurrence of anxiety symptoms at follow-up.

	**Bivariate**	**Multivariate**
Levels of UP (higher score = more intended)	1.03 (0.94 - 1.15)	1.18 (0.90 - 1.56)
Pregnancy outcome (0 = abortion)	1.27 (0.71 - 2.32)	2.53 (0.71 – 9.21)
Levels of UP x Pregnancy outcome	-	0.83 (0.60 - 1.14)
**Sociodemographic**		
Higher subjective SEP	0.92 (0.80 - 1.05)	na
Born outside the Netherlands	3.85 (1.22 - 11.55)*	na
Unemployed	3.25 (1.53 - 6.70)**	2.52 (1.11 - 5.54)*
Practically educated (vs theoretically)	0.63 (0.35 - 1.11)	na
Cohabiting	1.33 (0.72 - 2.57)	na
Age	1.02 (0.97 - 1.07)	na
Multiparous	1.37 (0.77 - 2.43)	na
Religious	1.52 (0.80 - 2.82)	na
**Abuse**		
Experiences with mental/physical abuse during youth	2.86 (1.42 - 5.61)***	na
Experiences with sexual abuse in lifetime	1.36 (0.68 - 2.59)	na
**History of anxiety/depression symptoms**	3.63 (2.00 - 6.77)***	2.88 (1.54 - 5.54)**
**Social support**		
Social support of family/friends	0.68 (0.47 - 0.99)*	0.73 (0.49 - 1.09)
Social support of partner	0.97 (0.77 - 1.25)	na
**Decision related**		
Perceived pressure	1.36 (0.90 - 1.99)	na
Difficulty	0.94 (0.75 – 1.19)	na
Certainty	1.26 (0.82 – 2.01)	na
Negative emotions	1.43 (1.09 - 1.87)**	1.55 (1.10 – 2.20)*

Notes. • p<.10, *p<.05, ** p<.01, *** p<.001. Na = not applicable, as the variable was excluded in the analysis. Variables were not included in the multivariate model if they were not related to the outcome in bivariate analyses (*p* < .10), or the model fit significantly approved when the variable was excluded from the multivariate model. All characteristics were measured at baseline.

Table S3b. Odds ratios (and 95% confidence intervals) from bivariate and multivariate logistic regression analyses examining the associations between selected characteristics and the risk on occurrence of depressive symptoms at follow-up.

	**Bivariate**	**Multivariate**
Levels of UP (higher score = more intended)	1.01 (0.91 - 1.12)	0.90 (0.68 - 1.17)
Pregnancy outcome (0 = abortion)	1.00 (0.57 - 1.77)	0.68 (0.17 - 2.59)
Levels of UP x Pregnancy outcome	-	1.21 (0.88 - 1.70)
**Sociodemographic**		
Higher subjective SEP	0.84 (0.74 - 0.97)*	0.88 (0.75 - 1.03) •
Born outside the Netherlands	2.63 (0.78 -7.94) •	na
Unemployed	2.64 (1.23 - 5.47)**	na
Practically educated (vs theoretically)	0.93 (0.53 - 1.64)	na
Cohabiting	1.30 (0.71 - 2.46)	na
Age	1.03 (0.98 - 1.08)	na
Multiparous	1.29 (0.73 - 2.26)	na
Religious	1.26 (0.66 - 2.34)	na
**Abuse**		
Experiences with mental/physical abuse during youth	2.30 (1.13 - 4.52)*	na
Experiences with sexual abuse in lifetime	2.14 (1.13 - 3.97)*	na
**History of anxiety/depression symptoms**	4.46 (2.49 - 8.24)***	5.37 (2.76 - 10.90)***
**Social support**		
Social support of family/friends	0.63 (0.44 - 0.90)*	0.66 (0.44 - 0.99)*
Social support of partner	.80 (0.65 - 1.00)*	na
**Decision related**		
Perceived pressure	1.36 (0.91 - 1.99)	na
Difficulty	1.15 (0.92 – 1.42)	na
Certainty	1.16 (0.76 – 1.81)	na
Negative emotions	1.33 (1.01 - 1.73)*	1.37 (0.95 - 1.97) •

Notes. • p<.10, *p<.05, ** p<.01, *** p<.001. Na = not applicable, as the variable was excluded in the analysis. Variables were not included in the multivariate model if they were not related to the outcome in bivariate analyses (*p* < .10), or the model fit significantly approved when the variable was excluded from the multivariate model. All characteristics were measured at baseline.
